# Pattern Changes in the Heart Rate Variability of Patients Undergoing Coronary Artery Bypass Grafting Surgery

**DOI:** 10.1155/2022/1455025

**Published:** 2022-04-30

**Authors:** Ngo Van Thanh, Nguyen Sinh Hien, Pham Nguyen Son, Pham Truong Son

**Affiliations:** ^1^Hanoi Heart Hospital, 92 Tran Hung Dao, Hoan Kiem District, Hanoi 11019, Vietnam; ^2^108 Military Central Hospital, 1B Tran Hung Dao, Hai Ba Trung District, Hanoi 11600, Vietnam; ^3^Heart Institute, 108 Military Central Hospital, 1B Tran Hung Dao, Hai Ba Trung District, Hanoi 11600, Vietnam

## Abstract

**Introduction:**

Coronary artery bypass grafting (CABG) with extracorporeal circulation is a key therapy for coronary artery disease (CAD). However, cardiovascular events and cardiac arrhythmias may still occur in these patients following surgery. Many studies have demonstrated a correlation between cardiac arrhythmias and heart rate variability (HRV). This study aimed to establish the temporal change pattern of HRV observed following CABG.

**Methods:**

A prospective method was used to study 119 consecutive patients with stable CAD who were assessed using 24-hour Holter recordings 2 days before CABG and 1 week, 3 months, and 6 months after the surgery at Hanoi Heart Hospital from June 2016 to August 2018. *Main results:* All the time-domain and frequency-domain parameters of HRV decreased precipitately after CABG and were mostly recovered 3 months postoperatively. The percentage of decreased HRV before surgery was 28.6% and 51.8% after 7 days, 19.6% after 3 months, and 12.7% after 6 months. ASDNN and SDNN before and after surgery had the highest rates of change.

**Conclusion:**

The early decrease in HRV observed 7 days after CABG may be related to the acute effects of the surgery. The recovery of HRV at 3 months after surgery, regardless of the preoperative state of the patients, implies that the autonomic nervous system (ANS) disorder may be improved at this time. At 6 months after surgery, the autonomic nervous injury was recovered in combination with improvement of reperfusion, resulting in improvement in almost all HRV indices compared with those indices preoperatively.

## 1. Introduction 

Coronary artery disease (CAD) is a common disease and the leading cause of death worldwide [1, 2]. Currently, coronary artery bypass grafting (CABG) with extracorporeal circulation is a key therapy for CAD; however, cardiovascular events and cardiac arrhythmias still occur in these patients after surgery [3–6]. Cardiac arrhythmias commonly occurring after surgery include atrial fibrillation (5–40%), ventricular tachycardia (26.6%), and ventricular fibrillation (2.7%) [7–9]. Cardiac arrhythmias account for 30–50% of all postoperative deaths [10]. Among the cardiac arrhythmias mentioned above, only 5–10% can be detected by a 12-lead electrocardiogram (12-lead ECG); this can be increased to up to 40–60% by using 24-hour Holter monitoring (or 24-hour Holter ECG) detection [11]. The autonomic nervous system (ANS) is a risk factor for the development of arrhythmias [12]. Holter ECG plays a direct role in assessing arrhythmias and an indirect role in assessing ANS activity through HRV, which is considered to be a predictor of arrhythmia and cardiovascular events [12, 13].

Several studies have evaluated HRV pre- and postoperatively. However, in these studies, HRV after CABG was only monitored at one time [14] or was assessed by sequential changes with a limited number of cases [15–17]. In this study, we assessed HRV in time-domain and frequency-domain by 24-hour Holter ECG in patients before and after CABG at 1 week, 3 months, and 6 months. The assessment of HRV 3 times post-surgery will help to predict ventricular arrhythmias, especially new-onset atrial fibrillation for acute post-surgery and stable stage after surgery, which may provide better prognosis and treatment in different postoperative periods.

### 1.1. Selection Criteria

A total of 119 patients with stable CAD in sinus rhythm undergoing isolated CABG surgery at Hanoi Heart Hospital between June 2016 and August 2018 were enrolled in this study.

### 1.2. Exclusion Criteria

Patients with an acute coronary syndrome, acute heart failure, or other acute diseases; patients with medical conditions where HRV could not be assessed before surgery, such as atrial fibrillation, frequent premature ventricular complex, sinus node dysfunction, and second and third-degree atrioventricular block; patients with pacemakers; patients with a congenital heart disease or combined cardiac surgeries; and patients who did not consent to participate in the study were excluded.

## 2. Methods and Research

### 2.1. Study Design

This study was conducted by prospective and descriptive methods and was approved by the Hospital Ethics Committee. Patients were advised of the protocol for the study and written informed consent was obtained.

### 2.2. Research Tools

Holter 24-hour recordings were made by 3-channel SEER LIGHTS Digital Holter recorders; an MSC 8800 Holter Monitoring system with Medical System International software version 5.02 was used.

### 2.3. Research Steps

#### 2.3.1. Perioperative Management

No patient took amiodarone preoperatively. Most patients were extubated on the day of surgery and oral medication was initiated on postoperative day 1, no inotropes were used at 3 days postoperatively. We tried to avoid prescribing drugs that affect HRV (calcium channel blockers, beta-blockers, antiarrhythmic drugs, etc.). If drugs affecting HRV were prescribed for preoperative treatment, we overcame this influencing factor by continuing its use after surgery.

#### 2.3.2. Holter ECG Recordings and HRV Analysis

The first recording was made 2 days before surgery, the second was made 7 days after surgery, the third was made 3 months after surgery, and the 4th was made 6 months after surgery. HRV was analyzed using the Holter monitoring system and then over-read manually. It was analyzed in Holter ECG recordings only in patients with a sinus rhythm. Holter ECG results were reviewed by a single cardiologist and only recordings with less than 15% of ectopic beats were used. All artifacts were cleaned and beats were modified as needed.

### 2.4. Evaluation Criteria

Most of the variables were calculated as recommended by the Task Force of the European Society of Cardiology and the North American Society of Pacing and Electrophysiology [18]. For frequency-domain analysis, the indices measured included VLF (the magnitude of the HRV in the VLF range, 0.0033 to 0.04 Hz), LF (the magnitude of the HRV in the LF range, 0.04 to 0.15 Hz), and HF (the magnitude of the HRV in the HR range, 0.15 to 0.04 Hz). HRV in the time-domain covered ASDNN (the mean of the standard deviations of all normal to normal intervals for 5 minutes of recording), rMSSD (the square root of the mean of the sum of the squares of differences between adjacent normal to normal intervals), pNN50 (percentage of normal to normal intervals >50 ms), SDNN (standard deviation of all normal to normal intervals), and SDANN (standard deviation of averages of normal to normal intervals in all 5-minute segments of recordings). Decreased HRV was defined according to Crawford et al. [19], i.e., if there was at least one index of expression upper to the limit level [19]. Atrial fibrillation was defined as at least 6 minutes of an irregularly irregular pattern of QRS waves recorded [19].

### 2.5. Statistical Analysis

The corrected data were processed, and HRV was computed. The results are expressed by mean value ± standard deviation with a minimum and maximum value. Normality of distribution of the variables was checked using the Kolmogorov–Smirnov test. Variables that are normally distributed have a histogram (or “density function”) that is bell-shaped, with only one peak, and is symmetric around the mean. Comparisons in parameters between two groups were performed using Pearson's X2 test for categorical variables, unpaired *t*-test for normally distributed variables, and Mann–Whitney's *U* test for skewed variables. Continuous variables underwent Student's unequal variance unpaired *t*-tests or nonparametric tests, as appropriate. All analyses were performed with the SPSS statistical package, version 11.0.

## 3. Results and Discussion

### 3.1. General Characteristics of the Patients

**Figure 1:**
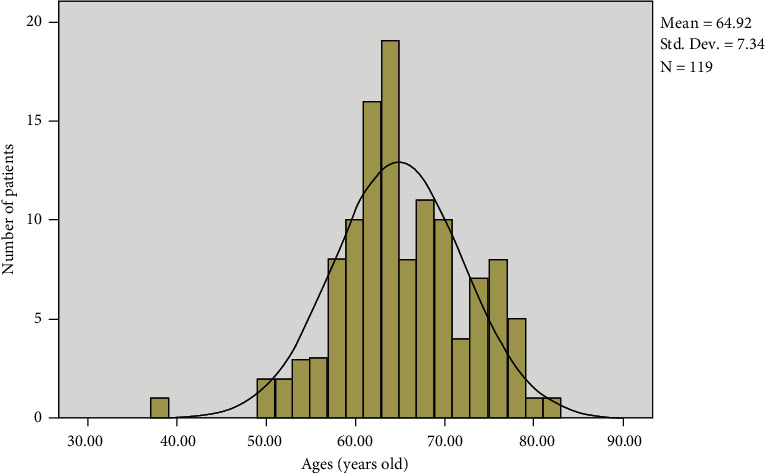
Age distribution.

A total of 119 patients were recruited for this study. The general characteristics of the studied patients are shown in Table 1 and Figure 1. The average age of these patients was 64.92 ± 7.34 years old, with the common age ranging between 60 and 70 years. The youngest patient was 38 years old and the oldest was 81 years old. The age of patients in this study is similar to that in published studies [20].

Males accounted for 83.2% of the patients (99 patients) and females accounted for 16.8% (20 patients). The proportion of men was more than four times that of women. This rate is similar to that of several published studies [21–23]. Generally, men are three to four times more likely to develop coronary heart diseases than women. The mean BMI of the patients was 22.99 ± 2.85 (ranging between 15.99 and 30.8). Being under/overweight or obese increases the frequency of pneumonia and it can be difficult to wean such patients off mechanical ventilation in the early stages of surgery [24]. Hypertension-related diseases were prevalent in 103 of the patients. These characteristics reflect the common risk factors of CAD such as advanced age, multiple risk factors, and comorbidities.

### 3.2. HRV in the Frequency Domain

All 119 patients had sinus rhythm Holter ECG results before surgery. After 7 days of CABG surgery, there were two cases of death and eight cases of persistent atrial fibrillation. At 3 months and 6 months postoperatively, there were 14 cases of atrial fibrillation and one more death, respectively. HRV is the oscillation of the heart rate interval that reflects the interaction of factors regulating heart rate. Age, race, sex, physical status, and concomitant diseases influence HRV. However, HRV in 24 hours is stable on a day-to-day, day-to-week basis in the absence of therapeutic intervention or major events and is regulated by the ANS [25]. Imbalances in the ANS have been shown to increase the risk of arrhythmia in CAD patients. Increments in the sympathetic nervous system (SNS) activity in CAD patients may lead to greater myocardial ischemia, which in turn, increases the SNS activity while decreasing the parasympathetic nervous system (PSNS) activity [25, 26].

Table 2 shows that the measured value of HRV according to the frequency domain changed over the study period. After 7 days of CABG surgery, the values of the frequency spectrum indices (VLF, LF, HF) were lower than before surgery; however, the LF/HF ratio remained unchanged. This is likely explained by acute postoperative injuries affecting the ANS, which decreased both the SNS (decreased VLF and LF) and PSNS (decreased HF) and continued the imbalance between the SNS and PSNS (LF/HF remained unchanged). Cheng et al. [15] tested 14 patients, and the result showed that total power and only low-frequency power (SNS activity) decreased significantly 1 month after CABG and returned to preoperative levels from the second month. There were no significant differences in high-frequency power (PSNS activity) and low-/high-frequency power ratio (SNS activity) before and after CABG. The author suggested that the CABG effect SNS and its improvement after 2 months of operation was attributed to the recovery from direct injury to the vagus nerve or sinus node. In the study with 22 patients, Brown et al. [16] found that overtime between six and 12 weeks postoperatively the indices of PSNS (HF/total power) improved significantly, whereas no change was seen in the LF/HF ratio. Both authors mentioned above found that either SNS or PSNS was affected by CABG. In our study, both SNS and PSNS were reduced at one week postoperatively and changed during the follow-up period. The difference maybe explained that in these previous studies, a limited case recruited can cause bias in statistical analysis and the difference in baseline characteristics maybe the reason.

3 months after surgery (Table 2), all values of the frequency domain indices had risen again and were not significantly different from the preoperative values (*p* (1–3) > 0.05), indicating the improved recovery of the ANS compared to 7 days after surgery due to the improvement of acute injury 3 months postoperatively. However, the LF/HF index was not improved, which implied a remaining SNS/PSNS imbalance. After 6 months of surgery, a significant increase was seen in LF but not in HF, which resulted in significantly increased LF/HF and the improvement of SNS/PSNS balance. Probably, before surgery, LF was markedly reduced and only mild reduction was seen in HF; therefore, postoperative LF increased remarkably and an increase was also found in postoperative HF but without significance. This may explain the results of previous studies where cardiovascular events and cardiac arrhythmias at 6 months after surgery continued to improve compared to those 3 months postoperatively [27, 28]. In another study [15], HRV did not exceed the preoperative level over 6 months after CABG, and the author suggested that there was an incomplete recovery from direct injury to the vagal nerve or sinus node or that revascularization due to CABG cannot improve the autonomic nervous activity of the heart in 6 months. This present study was carried out recently with good conditions and facilities, which may partly explain the better revascularization with the improvement of HRV as compared to the previous study.

### 3.3. HRV in the Time Domain

In this study, the time-domain indices of HRV also changed over time. After 7 days of surgery, all of the time domain indexes (ASDNN, rMSSD, pNN50, SDNN, SDANN, and mean NN) were lower than before surgery (Table 3). This shows that the acute effect of CABG surgery reduces the time-domain indices, which is similar to the results of the frequency-domain indices analysis above. Acute injuries caused by surgery include myocardium damage, autonomic nerve fibers (caused by cutting, burning, contusion, etc.), neurohumoral dilution due to cardiopulmonary system filling, bleeding, and fluid infusion [29,30]. The HRV indexes reflect the ANS, in which the SDNN index is governed by the SNS (like the LF index). The pNN50 and rMSSD indices are governed by the PSNS (like the HF index). In the first week after surgery, other authors have found that arrhythmias increase significantly compared to before surgery [31]. The decrease in the time-domain indices during this time might indirectly reflect postoperative damage, which may help predict postoperative arrhythmias. At 3 months after surgery, all HRV indexes in the time domain had increased and were equal to those before surgery (*p*(1–3) > 0.05, Table 3). HRV recovery at 3 months after surgery was consistent in both the frequency and time domains. This result further demonstrates that ANS damage (including the SNS and PSNS) caused by acute effects of CABG was generally healed 3 months after surgery. These results reach to an agreement with the other studies. Pedro Paulo S Soares [17] also noted that all the time-domain indexes of HRV decreased at 3 days, 6 days, and 15 days after CABG, which gradually increased at 30 days postoperatively. At 60 days and 90 days after surgery, all time domain indexes returned to pre-surgery values with no significant difference.

In this study, at 6 months after surgery, the ASDNN, SDNN, and SDANN indexes were higher than before surgery (Table 3), which is similar to the VLF and LF indices in the results of the analysis of the frequency domain indices, indicating an increased function of the SNS on the heart. Whereas, at 6 months, the rMSSD and HF indices changed insignificantly compared to those before and 3 months after surgery. Both indexes were controlled by the PSNS and depended on concomitant diseases such as hypertension, kidney failure, diabetes, and CAD. Despite reperfusion after surgery, the underlying disease still existed and a proportion of patients had not been completely reperfused, which may explain the incomplete recovery of the PSNS indicators with no significantly different values compared to those before surgery.

### 3.4. Decreased HRV

**Figure 2:**
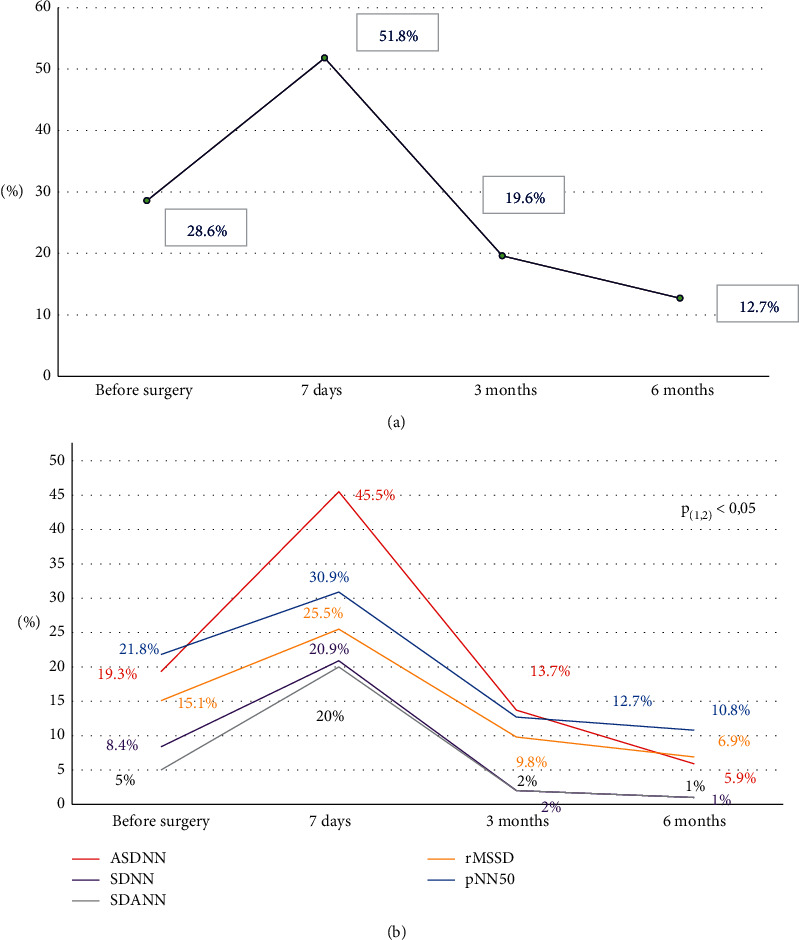
Heart rate variability. (a) Ratio of decreased heart rate variability before and after surgery and (b) ratio of decreased heart rate variability by each index.

In our study, there was a change in the percentage of decreased HRV at the following time points: before surgery (28.6%), 7 days after surgery (51.8%), 3 months after surgery (19.6%), and 6 months after surgery (12.7%). There was a sharply increased ratio of decreased HRV at 7 days postoperatively. Among these indices, the ratios of decreased ASDNN, SDNN, and SDANN were found to be significantly higher; this reflects the reduction of SNS function (Table 4, Figure 2). No significant changes in the ratio of decreased rMSSD or pNN 50 were observed, indicating that PSNS activity was not greatly affected.

The high proportion of patients with decreased HRV immediately after surgery was possibly due to the acute impact of the surgery, which reduced the HRV indices governed by the SNS, while the HRV indices reflecting the PSNS were not affected. Previous studies have shown that only a reduction in PSNS function is valuable in predicting cardiovascular events [32]. This may be the reason why a decreased HRV After 7 days of surgery has little value in predicting cardiovascular events. Likewise, regarding the consequences of decreased HRV in CABG patients with stable CAD, most authors have the same opinions. Milicevic et al., studying 175 CAD patients (124 with acute myocardial infarctions (MI) and 51 undergoing CABG surgery), showed that a decreased HRV in the CABG surgery group had a less predictive value for mortality than in the MI group [28]. Lakusic [29] also suggested that unlike a reduction of AMI in patients with MI, a reduction in HRV after CABG was not associated with major cardiovascular events [29]. Park et al. stated that a decreased HRV preoperatively has a predictive value in the occurrence of new atrial fibrillation and stroke following CABG [30].

In this study, decreased HRV was gradually recovered by the third month, in conjunction with significantly lower ratios of decreased HRV by each time-domain index. However, no significantly different value was observed compared to the preoperative values. By the sixth month, the ratios of decreased HRV by each index continued to decrease compared to the third month and a significant difference was observed compared to the pre-operative values, except for that of SDANN. By the sixth month, the HRV indices reflecting the PSNS and the indices reflecting the SNS and PSNS were significantly improved. The postoperative ANS injury which occurred during the first week lasted up to 3 months after surgery. By the sixth month, these damages had recovered in combination with the recovery of reperfusion, which resulted in an improvement in almost all HRV indices compared with the preoperative indices [31]. Despite a significant improvement of HRV comparing to pre-operation, a number of patients still have a decreased HRV, and HRV had not totally normalized. This likely explains why cardiac arrhythmia often occurred in the first week and then decreased gradually after 1, 3, and 6 months in most of the patients [32–35].

## 4. Conclusion

Our study shows that frequency- and time-domain indices changed over time following CABG. HRV in both SNS and PSNS indices reduced remarkably after 7 days, stabilized after 3 months, and increased 6 months after surgery. The ratio of decreased HRV was the highest 7 days after surgery, in which the ratio of decreased ASDNN and SDNN showed the most change, and then lowered after 3 months and minimized after 6 months with significant reduction compared to pre-operation.

### 4.1. Limitation

In this study, HRV was not assessed monthly during the follow-up; the pattern of change was only evaluated at 7 days, 3 months, and 6 months postoperatively. Cardiac events and death had not been followed up to find the relation with HRV change, which cannot help to provide prognosis. Due to lack of a large number of patients, HRV had not been analyzed in terms of baseline characteristics and surgery-related factors.

## Figures and Tables

**Table 1:** General characteristics, risk factors, and comorbidities.

Value parameters	Patients (*n* = 119)	
	Values (*n*)	Ratio (%)
Male	99	83.2
Smoking	55	46.2
BMI ≥ 23	61	51.2
History of myocardial infarction	10	8.4
Hypertension	103	86.6
Dyslipidemia	62	52.1
Chronic obstructive pulmonary disease (COPD)	4	3.4
Type 2 diabetes	40	33.6
Peripheral arterial disease	15	12.6
Renal failure ≥ IIIa	56	47.1

Age (years)	64.92 ± 7.34 (39–81)	
BMI (kg/m^2^)	22.99 ± 2.85 (15.99–30.8)	
Euro SCORE II (%)	1.31 ± 0.82 (0.6–4.9)	

**Table 2:** Heart rate variability by frequency domain before and after surgery.

Frequency domain		Timing			
		Before surgery ^(1)^ (*n* = 119)	After 7 days ^(2)^ (*n* = 109)	After 3 months ^(3)^ (*n* = 102)	After 6 months ^(4)^ (*n* = 102)
VLF (ms^2^)	($\bar{X}$ ± SD)	25.19 ± 12.28	18.32 ± 11.86	25.74 ± 9.18	29.75 ± 11.33
	*P*		*p*_(1-2)_ < 0.001	*p*_(1–3)_ > 0.05	*p*_(1–4)_ < 0.05

LF (ms^2^)	($\bar{X}$ ± SD)	16.26 ± 12.33	12.95 ± 11.93	17.06 ± 9.09	20.25 ± 9.91
	*P*		*p*_(1-2)_ < 0.05	p_(1–3)_ > 0.05	*p*_(1–4)_ < 0.05

HF (ms^2^)	($\bar{X}$ ± SD)	11.35 ± 7.21	8.74 ± 6.19	12.00 ± 6.26	12.91 ± 5.40
	*P*		*p*_(1-2)_ < 0.001	p_(1–3)_ > 0.05	*p*_(1–4)_ > 0.05

LF/HF	($\bar{X}$ ± SD)	1.43 ± 0.40	1.48 ± 0.64	1.48 ± 0.42	1.59 ± 0.43
	*P*		*p*_(1-2)_ > 0.05	p_(1–3)_ > 0.05	*p*_(1–4)_ < 0.05

**Table 3:** Heart rate variability (HRV) in time domain before and after surgery.

Time domain		Timing			
		Before surgery ^(1)^ (*n* = 119)	After 7 days ^(2)^ (*n* = 109)	After 3 months ^(3)^ (*n* = 102)	After 6 months ^(4)^ (*n* = 102)
ASDNN (ms)	($\bar{X}$ ± SD)	44.84 ± 20.14	34.54 ± 21.24	46.13 ± 16.53	52.23 ± 16.56
	*P*		*p*_(1-2)_ < 0.001	*p*_(1–3)_ > 0.05	*p*_(1–4)_ < 0.05

rMSSD (ms^2^)	($\bar{X}$ ± SD)	26.73 ± 12.15	22.14 ± 12.82	27.83 ± 12.18	29.14 ± 10.01
	*p*		*p*_(1-2)_ = 0.001	*p*_(1–3)_ > 0.05	*p*_(1–4)_ > 0.05

pNN 50 (%)	($\bar{X}$ ± SD)	6.84 ± 7.24	4.94 ± 8.78	7.69 ± 7.74	8.40 ± 6.72
	*p*		*p*_(1-2)_ < 0.05	*p*_(1–3)_ > 0.05	*p*_(1–4)_ > 0.05

SDNN (ms)	($\bar{X}$ ± SD)	101.18 ± 34.28	76.65 ± 35.04	107.5 ± 27.27	121.5 ± 25.98
	*P*		*p*_(1-2)_ < 0.001	*p*_(1–3)_ > 0.05	*p*_(1–4)_ < 0.001

**Table 4:** Ratio of decreased heart rate variability before and after surgery.

Time domain		Timing			
		Before surgery ^(1)^ (*n* = 119)	After 7 days ^(2)^ (*n* = 109)	After 3 months ^(3)^ (*n* = 102)	After 6 months ^(4)^ (*n* = 102)
Decreased heart rate variability (*n*, %)	Yes	34 (28.6)	57 (52.3)	20 (19.6)	13 (12.7)
	No	85 (71.4)	52 (47,7)	82 (80,4)	89 (87.3)
			*p*_(1,2)_ < 0,001	*p*_(1,3)_ < 0.05	*p*_(1,4)_ < 0.05

ASDNN (ms)	<30	23 (19.3)	50 (45.9)	14 (13.7)	6 (5.9)
	≥30	96 (80.7)	59 (54.1)	88 (86.3)	96 (94.1)
	*p*		*p*_(1,2)_ < 0.05	*p*_(1,3)_ > 0.05	*p*_(1,4)_ < 0.05

rMSSD (ms^2^)	<15	18 (15.1)	28 (25.7)	10 (9.8)	7 (6.9)
	≥15	101 (84.9)	81 (74.3)	92 (90.2)	95 (93.1)
	*p*		*p*_(1,2)_ > 0.05	*p*_(1,3)_ > 0.05	*p*_(1,4)_ < 0.05

pNN 50 (%)	<0,75	26 (21.8)	34 (31.2)	13 (12.7)	11 (10.8)
		93 (78.2)	75 (68.8)	89 (87.3)	91 (89.2)
	*p*		*p*_(1,2)_ > 0.05	*p*_(1,3)_ > 0.05	*p*_(1,4)_ < 0.05

SDNN (ms)	<50	10 (8.4)	23 (21,1)	2 (2.0)	1 (1.0)
		109 (91.6)	86 (78.9)	100 (98.0)	101 (99.0)
	*p*		*p*_(1,2)_ < 0.05	*p*_(1,3)_ < 0.05	*p*_(1,4)_ < 0.05

SDANN (ms)	<40	6 (5.0)	22 (20.2)	2 (2.0)	1 (1.0)
		113 (95.0)	87 (79.8)	100 (98.0)	101 (99.0)
	*p*		*p*_(1,2)_ < 0.05	*p*_(1,3)_ > 0.05	*p*_(1,4)_ > 0.05

## Data Availability

The data used to support the findings of this study can be made available on request through e-mail to the corresponding author.
